# Potent and Specific Inhibition of Glycosidases by Small Artificial Binding Proteins (Affitins)

**DOI:** 10.1371/journal.pone.0097438

**Published:** 2014-05-13

**Authors:** Agustín Correa, Sabino Pacheco, Ariel E. Mechaly, Gonzalo Obal, Ghislaine Béhar, Barbara Mouratou, Pablo Oppezzo, Pedro M. Alzari, Frédéric Pecorari

**Affiliations:** 1 Institut Pasteur de Montevideo, Recombinant Protein Unit, Montevideo, Uruguay; 2 Institut Pasteur, Unité de Microbiologie Structurale, CNRS UMR 3528, Paris, France; 3 INSERM UMR 892 - CRCNA, Nantes, France; 4 CNRS UMR 6299, Nantes, France; 5 University of Nantes, Nantes, France; 6 Institut Pasteur de Montevideo, Protein Biophysics Unit, Montevideo, Uruguay; Centro Nacional de Biotecnologia - CSIC, Spain

## Abstract

Glycosidases are associated with various human diseases. The development of efficient and specific inhibitors may provide powerful tools to modulate their activity. However, achieving high selectivity is a major challenge given that glycosidases with different functions can have similar enzymatic mechanisms and active-site architectures. As an alternative approach to small-chemical compounds, proteinaceous inhibitors might provide a better specificity by involving a larger surface area of interaction. We report here the design and characterization of proteinaceous inhibitors that specifically target endoglycosidases representative of the two major mechanistic classes; retaining and inverting glycosidases. These inhibitors consist of artificial affinity proteins, Affitins, selected against the thermophilic CelD from *Clostridium thermocellum* and lysozyme from hen egg. They were obtained from libraries of Sac7d variants, which involve either the randomization of a surface or the randomization of a surface and an artificially-extended loop. Glycosidase binders exhibited affinities in the nanomolar range with no cross-recognition, with efficient inhibition of lysozyme (K_i_ = 45 nM) and CelD (K_i_ = 95 and 111 nM), high expression yields in *Escherichia coli*, solubility, and thermal stabilities up to 81.1°C. The crystal structures of glycosidase-Affitin complexes validate our library designs. We observed that Affitins prevented substrate access by two modes of binding; covering or penetrating the catalytic site *via* the extended loop. In addition, Affitins formed salt-bridges with residues essential for enzymatic activity. These results lead us to propose the use of Affitins as versatile selective glycosidase inhibitors and, potentially, as enzymatic inhibitors in general.

## Introduction

Glycosidases are involved in a variety of metabolic disorders and human diseases such as type II diabetes, Gaucher disease, cancers and asthma [1], [2], [3], [4]. They are thus actively studied not only to probe their functions, but also as targets for inhibitor drugs to treat human diseases. However, achieving specific and efficient inhibition of a particular glycosidase represents a major challenge because a given organism can produce many different glycosidases, and also because this class of enzymes has evolved different functional specificities from a single structural scaffold, giving rise to similar active-site architectures and catalytic mechanisms. *In vivo*, a lack of selectivity for a drug can increase the risk of undesirable effects or even lead to toxicity [5] by off-target effects.

The use of small-molecular weight compounds is a powerful approach to modulate the activity of individual glycosidases [6], and a number of small-molecule inhibitors have been described for these enzymes. Although this class of inhibitors is attractive for the development of drugs, they can interact with non-target proteins and thus few high-quality inhibitors useful for therapy have been reported (for a review see refs. [6] and [7]). An alternative strategy is the development of proteinaceous inhibitors. Compared to small-molecule ligand-protein interactions, protein-protein or protein-antibody interactions generally involve much larger interfaces (typically 800–1000 Å^2^, [8], [9], [10]), a favorable feature to achieve binding with high specificity and selectivity. Antibodies can bind quite different compounds specifically, but it may be difficult to obtain candidates that bind a cleft-shaped active site [11], such as those of endo-glycosidases. Alternatives to classic antibodies have emerged based on immunoglobulin or non-immunoglobulin folds (for a review see refs. [12] and [13]) to derive specific binders of targeted proteins. Only a few of these binders have been shown to be potent enzymatic inhibitors and even fewer have been described at the structural level to understand their mechanisms of inhibition. As examples, the Ecotin scaffold has been used to generate a highly specific inhibitor of the protease kallikrein with a K_i_ of 11 pM [14] while binders with inhibition properties for hen egg white lysozyme (HEWL) have been derived from various proteins (VHH, shark IgNAR and an anticodon recognition domain of the aspartyl tRNA synthetase), and have been structurally described to mimic the oligosaccharide substrate of this glycosidase [11], [15], [16], [17], [18].

A general and convenient strategy to develop inhibitors would be to use a unique scaffold protein able to either cover or deeply penetrate active sites. The success of this approach depends essentially on the ability of the scaffold protein to recognize catalytic sites with different shapes. As an important step towards this goal, we have exploited the plasticity and stability of artificial 7 kDa affinity proteins (Affitins) [19], [20], [21], [22], [23] derived from extremophilic proteins, such as DNA-binding protein 7d (Sac7d), which are found in various Archaea such as *Sulfolobus*, *Acidianus*, and *Metallospharea* genera. With their small size and their low structural complexity, Affitins occupy an intermediate position between peptides and proteins. Previously, we reported that Affitins can bind different epitopes of the same target *via* two different modes of binding: one involving a flat surface and the other involving a flat surface and two short loops [23].

Based on these results, in this work we designed two Affitin libraries in which a loop of Sac7d was extended by four additional randomized residues. As a proof of concept that Affitins may inhibit different glycosidases specifically, we used these libraries (L3 and L4) and those we had previously designed without an extended loop (L1 and L2) to select Affitins specific for the inverting endo-glycosidase CelD from *Clostridium thermocellum* (EC 3.2.1.4). We also analyzed an Affitin specific for the well-studied (retaining endo-glycosidase) HEWL (EC 3.2.1.17) previously selected from the library L1 [20], [24]. These two glycosidases hydrolyze the O-glycosyl bond and are representative of the two main glycosidase mechanisms of action [25]. Isolated Affitins were shown to be potent inhibitors of CelD and of HEWL, with K_i_ in the nanomolar range, without cross-recognition. The crystal structures of Affitin-CelD and Affitin-HEWL complexes revealed their inhibition mechanisms, and provided useful hints for further inhibitor improvement. These results lead us to propose the use of Affitins as versatile and thermostable selective glycosidase inhibitors.

## Materials and Methods

Chemicals were purchased from Sigma-Aldrich. Enzymes and buffers for molecular biology were purchased from Thermo Scientific or New England Biolabs unless otherwise indicated. Oligonucleotides were purchased from Eurofins. All PCR were performed using Vent polymerase.

### Construction of Libraries and Selections

Since we have observed that two tryptophans at positions 8 and 9 can promote multimerization of Affitins, we either did not randomize these two positions (library L3) or limited their randomization using NHK codons (library L4) that do not encode tryptophan. This codon sub-set also excludes Gly, Cys and Arg. The other positions were randomized using NNS triplets that encode all amino acids and only one stop-codon.

The generation of libraries L1 and L2, which corresponds to the random mutagenesis of positions 7, 8, 9, 21, 22, 24, 26, 29, 31, 33, 40, 42, 44, and 46 and of positions 26, 27, 28, 29, 31, 42, 44, 46, 47, and 48, respectively, in Sac7d protein has been previously described [19], [23]. To construct library L3, which corresponds to the random mutagenesis of positions 7, 26, 27, 27a, 27b, 27c, 27d, 28, 29, 31, 44, 46, and 48 in Sac7d protein, the same protocol was used with the following oligonucleotides: T7B (5′- ATACGAAATTAATACGACTCACTATAGGGAGACCACAACGG-3′), T7C (5′-ATACGAAATTAATACGACTCACTATAGGGAGACCACAACGGTTTCCCTC-3′), SDA_MRGS (5′- AGACCACAACGGTTTCCCTCTAGAAATAATTTTGTTTAACTTTAAGAAGGAGATATATCCATGAGAGGATCG-3′), SClib2.1 (5′- GGAGATATATCCATGAGAGGATCGCATCACCATCACCATCACGGATCCGTCAAGGTGAAATTC-3′), SClib6.2.

(5′-GGATCCGTCAAGGTGAAATTCNNSTATAAAGGCGAAGAAAAAGAAGTGGACACTAGTAAGATC-3′), SClib8.3 (5′-CTTGCCGTTGTCGTCGTAGGTAAASNNCACSNNSNNSNNSNNSNNSNNSNNSNNACGCCAAACTTTCTTGATCTTACTAGTGTCCACTTC-3′), SClib6.4.

5′-TAATAACTCTTTCGGGGCATCSNNCTCSNNCACSNNGCCACGGCCGGTCTTGCCGTTGTCGTCGTAGG-3′), SClib2.5 (5′-CCATATAAAGCTTTTTCTCGCGTTCCGCACGCGCTAACATATCTAATAACTCTTTCGGGGCATC-3′), tolAk (5′-CCGCACACCAGTAAGGTGTGCGGTTTCAGTTGCCGCTTTCTTTCT-3′). To construct library L4 which corresponds to the random mutagenesis of positions 7, 8, 9, 26, 27, 27a, 27b, 27c, 27d, 28, 29, 31, 44, 46, and 48 in Sac7d protein, the same protocol was used but replacing SClib6.2 with SClib7.2 (5′-GGATCCGTCAAGGTGAAATTCNNSNHKNHKGGCGAAGAAAAAGAAGTGGACACTAGTAAGATC-3′). Both libraries were constructed in the ribosome display format with estimated numbers of independent variants of about 10^12^ [26].

The preparation of targets by *in vitro* biotinylation was performed as previously described [19], [23]. The ribosome display selections were also performed as previously described [26], except that the incubation time for the translation reaction was 10 min while the incubation times for the pre-panning and panning steps were 30 min in both cases. The RT-PCR was as follows: for selection rounds 1 and 2, an initial denaturation step at 95°C for 30 s, followed by 45 cycles of 30 s at 95°C, 30 s at 63°C, and 30 s at 72°C with a final elongation step of 5 min at 72°C. For selection rounds 3 and 4, it was the same program but with 40 cycles instead of 45. For the selections, 100 µl of biotinylated CelD (250 nM for round 1, 200 nM for round 2 and 150 nM for rounds 3 and 4) was bound on MaxiSorp ELISA plates (Nunc) previously coated with NeutrAvidin (Thermo Scientific) or streptavidin (Sigma-Aldrich), which were alternated during four or six selection rounds. To isolate high-affinity binders, the time in wash-steps was increased during the selection (6 washes of 30 s, 1 min, 3 min and 10 min for rounds 1, 2, 3 and 4, respectively).

### Analysis of Selected Pools and Isolated Clones

To assess enrichments of the selections, the output RNA obtained after four or five rounds of selection were translated *in vitro* and tested in MaxiSorp ELISA wells coated with streptavidin/NeutrAvidin and biotinylated CelD as previously described [23], [26]. A negative control was performed with wells coated with only streptavidin or NeutrAvidin. For anti-CelD Affitins, the RT-PCR product from L3 and L4 obtained after round 4, and which gave a positive signal in ELISA, was cloned into the *Bam*HI and *Hind*III restriction sites of the pFP1001 vector, and the ligation mixture was transformed into *E. coli* DH5αF’IQ (Invitrogen) for the isolation of individual clones [26]. The screening of individual clones was performed by ELISA as described before [23], [26].

### Production and Purification of Proteins

HEWL was obtained from a commercial source (Sigma-Aldrich). CelD glycosidase (residues 34 to 577) was cloned into the pQE80 vector, which introduced a TEV cleavage site and a His-tag at the N-terminal of the protein. CelD was expressed in *E. coli* M15pREP4 (Qiagen). Affitins previously selected as in the “Analysis of selected pools and isolated clones” section, were expressed on a large scale. All cultures were grown to reach an OD_600_ of 1.2 in 2xYT and protein expression was induced with 0.5 mM IPTG for 16 h at 37°C for CelD and at 30°C for Affitins. Cells were pelleted by centrifugation at 4000 g for 15 min and resuspended in lysis buffer (50 mM NaPO_4 _pH 7.5, 500 mM NaCl, 20 mM imidazole, 1 mg/ml HEWL, except for the anti-HEWL binder where HEWL was omitted) and frozen at −80°C. Pellets were thawed, sonicated and centrifuged at 18,000 g for 45 min. After purification by immobilized metal ion affinity chromatography (IMAC), using Chelating Sepharose Fast Flow resin charged with Ni^2+^ (GE Healthcare), proteins were injected into a Superdex75 16/60 column (GE Healthcare) equilibrated with 40 mM Tris-HCl pH 7.7 for CelD or 25 mM Tris-HCl pH 8.0, 500 mM NaCl for Affitins. The His-tag of purified CelD was cleaved with TEV protease. Uncleaved proteins, His-tag peptides and TEV proteases were removed by a second IMAC purification step. Purified proteins were quantified spectrophotometrically at 280 nm according to their molar extinction coefficients. Finally, for ITC and DSC analyses, proteins were desalted to PBS by using a HiPrep 26/10 desalting column (GE Healthcare).

### Thermostability Measurements

DSC experiments were carried out in PBS, in a VP-DSC instrument (Microcal, Northampton, MA) and data analyzed with the software supplied with the equipment. The temperature was increased by 1°C per min from 30 to 120°C, and proteins were added at concentrations of 195, 217 and 300 µM for E12, H3 and H4 Affitins, respectively.

### Isothermal Titration Microcalorimetry

ITC experiments were conducted using a VP-ITC instrument (Microcal, Northampton, MA). Injections of 10 µl of the different Affitins were added from a computer–controlled microsyringe at intervals of 460 s into the sample solution containing CelD or HEWL under constant stirring (400 rpm) at 25°C. The concentrations used for the experiments were 9.5 µM for CelD; 6.5 µM for HEWL and 195, 217 and 157 µM for E12, H3 and H4 Affitins, respectively. Titrations were carried out in PBS buffer. Data analysis was performed using Origin7 (Microcal), after subtraction of a manually-corrected baseline generated using constant heat values at the end of titration. Binding isotherms were fitted to a simple 1∶1 Langmuir model. The same experiments were carried-out at 60°C for H3-CelD and E12-CelD.

### Enzymatic Inhibition Assays

CelD activity was determined by a colorimetric assay using ρ-nitrophenyl-β-D-cellobioside (p-NPC, Sigma-Aldrich) as substrate; 500 nM of CelD was incubated with 0.5 mM of substrate for 1 h at room temperature or 60°C in PBS. The color change was measured spectrophotometrically at 415 nm and the final value corresponded to 100% of relative activity. HEWL activity was determined by monitoring the change in turbidity at 450 nm of a suspension of *M. lysodeikticus* bacteria (Sigma-Aldrich) in 100 mM potassium phosphate buffer, pH 7.0, as reported in [11]. Briefly, 400 µg/ml of cells was incubated with 20 nM of HEWL at RT for 1 h and the absorbance was measured. Enzymatic inhibition assays were carried out with different molar ratios of enzyme: Affitin (1∶1, 1∶2, 1∶5 and 1∶10). For the determination of the K_i_ values of anti-CelD Affitins, inhibition was carried out in PBS at 25°C, in the presence of 200 nM of CelD for 35 min. The substrate concentration (p-NPC) was, 5, 3, 2, 1, 0.5, 0.2, 0.05 and 0.02 mM. The inhibitor concentration (E12 or H3) was 0, 20, 50, 100 and 200 nM. Experiments were carried out in triplicate and fitted to a competitive inhibition model for anti-CelD or “One site–Fit K_i_” for anti-HEWL Affitins using GraphPad Prism software (GraphPad Software).

### Crystallization of Complexes

Anti-CelD Affitins and CelD were mixed in a 2∶1 molar ratio to obtain a final concentration of 20 mg/ml for CelD in 25 mM Tris-HCl pH 8.0 and 100 mM NaCl. Affitin H4 and HEWL were mixed in a 1∶1 molar ratio and the complex was purified by gel filtration chromatography with a Superdex75 16/60 column, equilibrated with the same buffer. The purified complex was concentrated to 80 mg/ml before setup crystallization trials. A crystallization screening was performed by mixing the complex with 480 different buffers (1∶1) at 19°C using the hanging-drop vapor-diffusion method. The crystallization buffer for the HEWL-H4 complex was 20% PEG 8000 (w/v), 100 mM CAPS pH 10.5 and 200 mM NaCl. For anti-CelD Affitins, it was 100 mM HEPES pH 7.5, 10.4% PEG 8000 (w/v), and 500 mM calcium acetate. Crystals were frozen in 20% glycerol diluted with the crystallization buffer.

### Diffraction Data Collection

X-ray diffraction data for HEWL and CelD complexes were collected at the European Synchrotron Radiation Facility (ESRF) beamlines ID14–4 and ID23–2, respectively. Data reduction and scaling were performed with XDS [27] and Aimless [28], respectively.

### Structure Determination, Model Building and Refinement

Crystal structures of HEWL and CelD in complex with their respective Affitins were solved by molecular replacement using Phaser [29]. Partial molecular replacement solutions using either HEWL (PDB code, 1GWD) or CelD (PDB code, 1CLC) as search models displayed extra electron density readily interpretable as the Affitin chain, which was manually traced. The structures were refined with Buster [30] and alternating rounds of model rebuilding with Coot [31]. All models were subjected to a last round of anisotropic B-factor refinement with Refmac [32] before MolProbity [33] validation. All structural representations were prepared with Pymol [34]. Protein-protein interaction parameters were calculated using the PISA server (www.ebi.ac.uk/msd-srv/prot_int/pistart.html) and LIGPLOT [35]. Shape complementarity analysis was performed with the SC program included in the CCP4 suite using default settings [36].

### Accession Numbers

The atomic coordinates and structure factors have been deposited in the Protein Data Bank with the following accession codes: 4CJ0 (CelD-E12), 4CJ1 (CelD-H3) and 4CJ2 (HEWL-H4).

## Results

### Library Designs

**Figure 1:**
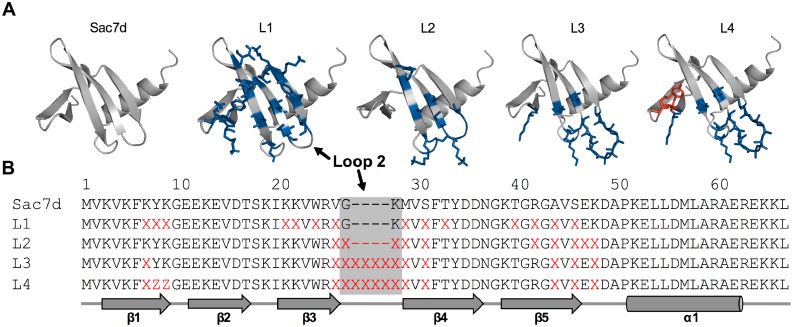
Schematic representation of Affitin libraries. (**A**) Sac7d wild-type structure. Two β-sheets composed of two (β_1_β_2_) and three (β_3_β_4_β_5_) antiparallel β-strands followed by an amphipathic α-helix. Randomized residues of designed libraries are shown in blue and red, and were mutated with NNS and NHK codons, respectively. The position of the randomized loop, extended or not, is labeled “Loop 2”. (**B**) Alignment of designed libraries. Secondary structure elements are indicated below the sequences. X represents all residues and Z all residues except Gly, Cys, Arg and Trp.

**Figure 2:**
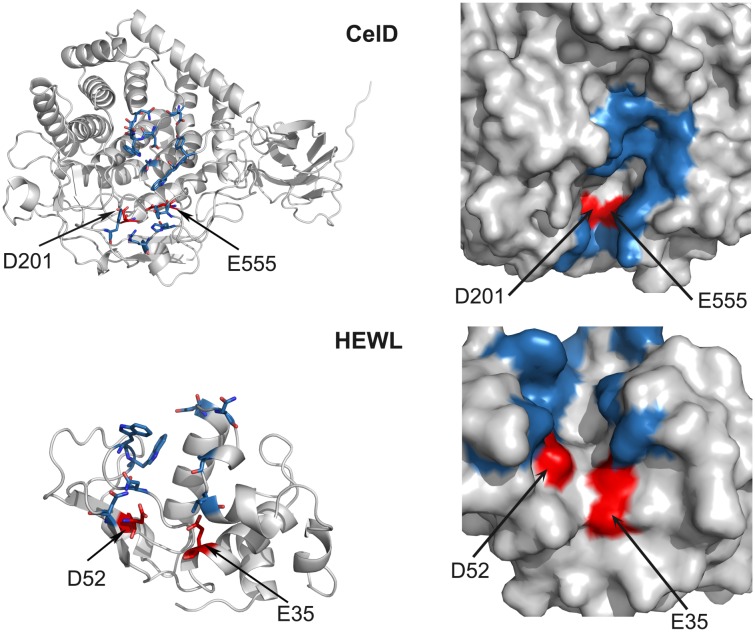
Crystal structures of CelD and HEWL glycosidases. Glycosidases (PDB codes: 4CJ1 and 4CJ2, respectively) are colored in gray with catalytic clefts in blue and catalytic residues in red. The right panel is a zoom view of active sites to show how catalytic residues are buried in CelD and less in HEWL.

Endo-glycosidases have cleft-shaped active sites and it is well known that loops can penetrate clefts. The short loop connecting β3-β4 strands (hereafter called “loop 2”) of Sac7d was demonstrated to participate in the recognition of human immunoglobulin in a previously isolated anti-IgG Affitin [23]. Thus, we investigated if an artificially-extended loop 2, with an additional four residues between Gly27 and Lys28, could mimic this binding mode (libraries L3 and L4, Figure 1A) and could be helpful for efficient enzymatic inhibition. For example, CelD has deeply buried catalytic residues (Figure 2).

### Selection of Anti-glycosidase Affitins

Two pools of libraries were constituted including the presence of the short (L1+L2) or long (L3+L4) loop 2, and selections were then performed in parallel by ribosome display using immobilized CelD as a target protein. For the L3+L4 selection, an ELISA after the fourth round indicated the expected enrichment in specific CelD binders. Two more rounds were performed for the L1+L2 selection without detectable enrichment.

### Characterization of Anti-glycosidase Binders

#### Sequence analysis expression and purification of selected binders

**Figure 3:**
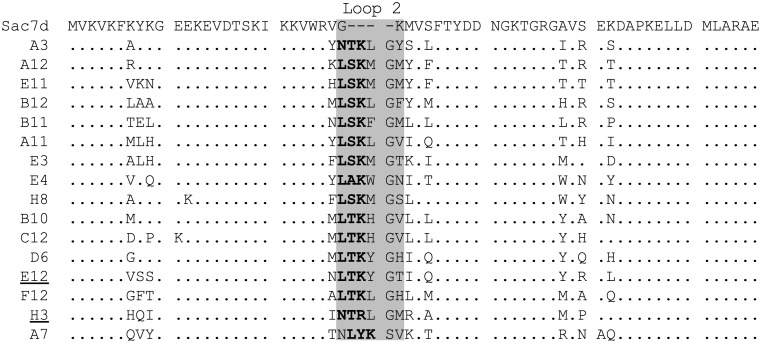
Sequence of anti-CelD Affitins. Alignment of the sixteen clones selected by ribosome display against the CelD enzyme. The conserved motif (Leu-Ser/Thr-Lys) inside the randomized and extended β-hairpin 2 is labeled in bold letters. Affitins studied in this work are underlined (E12 and H3).

Ninety-four randomly picked individual clones were screened by ELISA. Sixteen showed significant and specific CelD binding and were sequenced (Figure 3). Sequences originating from both libraries used for this selection (L3+L4) were identified. The 16 clones represented a variety of sequences. The motif Leu-Thr/Ser-Lys inside the extended loop was conserved, except in Affitin H3, where Leu was changed to Asn and Lys to Arg, although the latter implies a conservation of positive charge at this position. The other randomized positions did not show a conserved sequence. These data suggest that the extended loop might contribute significantly to the binding and highlight the probable importance of a positively charged residue.

**Figure 4:**
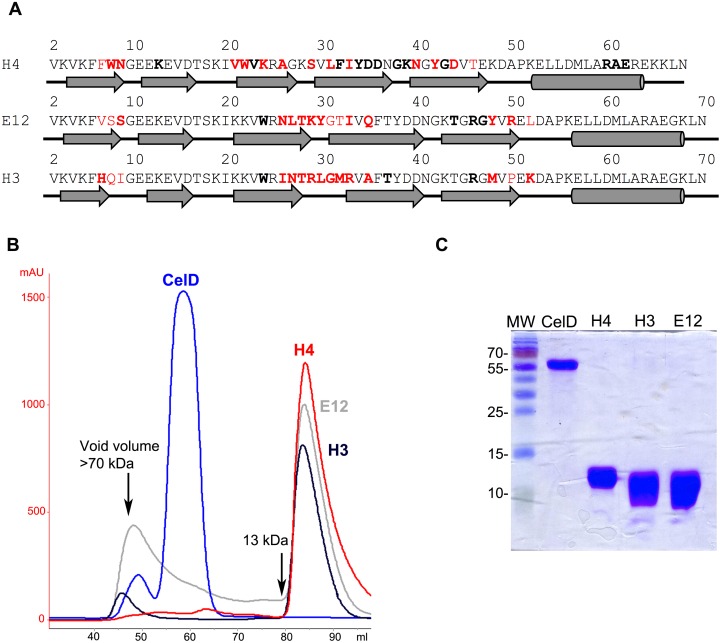
Sequences and production of anti-glycosidase Affitins. (**A**) Secondary structure elements according to crystallographic structure are shown below the sequences. Residues that were randomized are labeled in red. Residues that are involved in interaction with less than 10% of the buried surface appear in bold letters. (**B**) Size-exclusion purification of CelD (blue) and Affitin H4 (red), E12 (gray) and H3 (black) using a Superdex75 16/60 column. Arrows show the molecular weight obtained at the defined retention volumes. (**C**) SDS-PAGE 15% showing the final purity of CelD and the Affitins. Molecular weights are indicated in kDa.

With the aim of comparing potentially different modes of binding, we also included Affitin H4, which was selected against HEWL from the L1 library [20], and does not display obvious sequence similarity with anti-CelD Affitins (Figure 4A). Affitins with substantial sequence differences (E12 and H3) and Affitin H4 were produced in *E. coli* and purified to homogeneity (Figure 4B–C). These Affitins were predominantly eluted in size-exclusion chromatography at the volume corresponding to monomers. The production yields were up to 40 mg/L of shake flask culture (E12 and H3), and higher for H4 (90 mg/L culture).

#### Binding properties of anti-glycosidase variants

**Figure 5:**
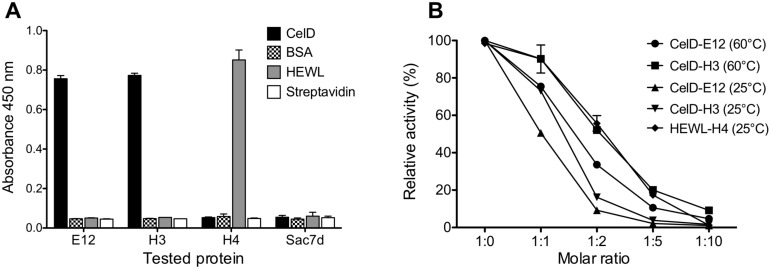
Biochemical properties of binders. (**A**) The interaction of E12, H3 and H4 Affitins (1 µM) was assayed by ELISA with immobilized CelD, streptavidin, BSA and HEWL. Sac7d wild-type was used as the negative control of binding at the same molar concentration. (**B**) Activity percentage of the thermophilic CelD glycosidase at 60°C and 25°C and of HEWL at 25°C. Different molar ratios (1∶1, 1∶2, 1∶5 and 1∶10) of Affitins were used as inhibitors.

The specificity of purified proteins was tested by ELISA analysis using the targets, streptavidin and bovine serum albumin (BSA) (Figure 5A). Affitins bound exclusively to their corresponding targets and not to unrelated proteins.

**Figure 6:**
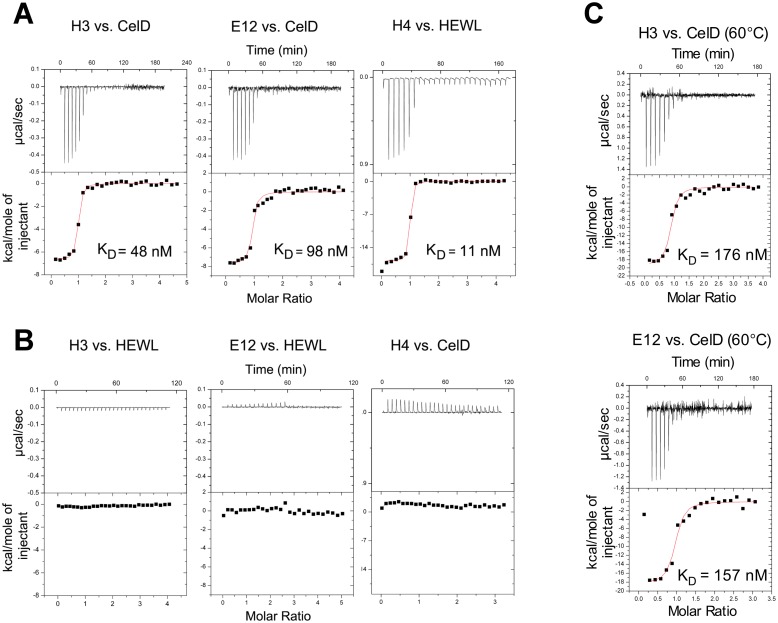
ITC analysis of anti-glycosidase binders. (**A**) ITC titrations at 25°C of Affitin H3 with CelD, Affitin E12 with CelD and Affitin H4 with HEWL. (**B**) Cross-recognitions were tested at 25°C for Affitin H3 with HEWL, Affitin E12 with HEWL and Affitin H4 with CelD. (**C**) ITC titrations at 60°C of Affitins H3 and E12 with CelD. The top panel for ITC shows data obtained from injections of Affitins while the bottom panel shows the integrated curve showing experimental points (filled squares) and the best fit (red line).

**Figure 7:**
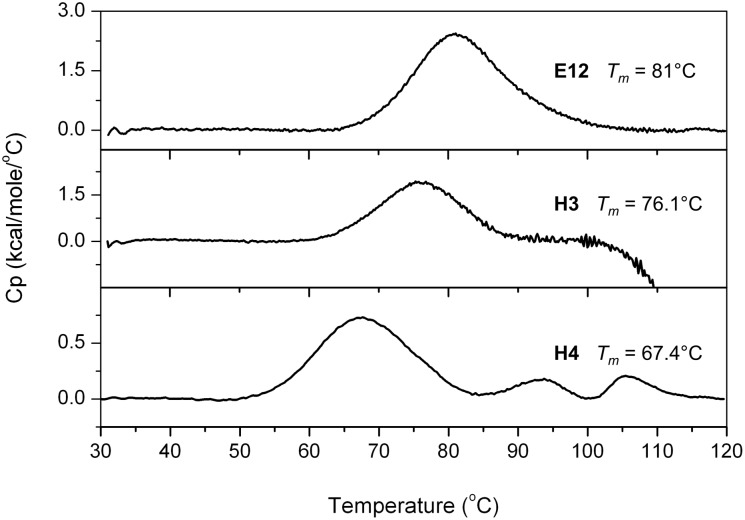
Thermal stabilities of anti-glycosidase binders. DSC curves of E12, H3 and H4 Affitins.

Isothermal titration microcalorimetry (ITC) binding analysis showed a stoichiometry close to 1, indicating a simple 1∶1 binding mode of interaction for all binders (Figure 6A, Table 1). Affinity values determined for E12 and H3 CelD binders were in the nanomolar range (98 nM and 48 nM, respectively), while the affinity value measured for H4 (11 nM) was similar to a value obtained by surface plasmon resonance analysis [20]. Since Affitins and CelD enzyme [37] are thermostable (Figure 7, Table 1), we also determined K_D_ values of anti-CelD binders at 60°C, the optimal temperature for the activity of CelD; they were 176 and 157 nM for H3 and E12, respectively (Figure 6C). These results indicate that, although these Affitins were selected at 4°C, they showed an ability to interact with CelD with high affinity over a wide temperature range.

**Table 1:** Binding affinity, thermal stability parameters and inhibition constants of anti-glycosidase Affitins.

Target, Affitin	*K*_D,_ nM^a^	Δ*G*, kcal/mol	Δ*H*, kcal/mol	*T*Δ*S*, kcal/mol	n	*K*_i_, nM	T*m*, °C^b^
HEWL, H4	11±2.8	−10.84	−16.77	−5.93	0.92	45±2	67.4
CelD, H3	48±10	−9.96	−6.69	3.27	0.93	111±10	76.1
CelD, E12	98±36	−9.56	−7.87	1.69	0.90	95±11	81.1

a Affinity obtained by ITC analysis at 25°C;

b Thermal melting obtained by DCS analysis. Errors shown derived from fitting to a 1∶1 binding model for the K_D_, and from a competitive inhibition or “One site–Fit” model for the K_i_.

Thermodynamic parameters of the interactions were determined and indicated a favorable enthalpy for all cases and favorable entropy for H3 and E12 at 25°C (Table 1). Affitin H4 showed an enthalpy-driven reaction with a higher favorable binding enthalpy and unfavorable binding entropy, consistent with Affitins that bind through mutagenesis on the same surface [19].

Finally, the binding of anti-HEWL onto CelD and the binding of anti-CelD Affitins onto HEWL were tested by ITC. No cross-recognition could be observed (Figure 6B) further supporting the ELISA results.

#### Thermostability of anti-glycosidase Affitins

Thermal stabilities of E12 and H3 Affitins determined by differential scanning calorimetry (DSC) analysis were of 81.1°C and 76.1°C, respectively (Figure 7, Table 1). These results confirm that the extension of loop 2 is compatible with thermally stable Affitins. DSC scans were characteristic of cooperative unfolding, indicating that variants were well folded. However, both Affitins exhibited different behavior at high temperature. Affitin E12 showed no sign of protein aggregation after T*_m_* was reached, while H3 showed an irreversible unfolding. H4 Affitin was also thermally stable and showed a primary T*_m_* of 67.4°C with unfolding intermediates at higher temperatures.

### Study of the Enzymatic Inhibition Properties of Affitins

The inhibitory properties of the purified Affitins were first analyzed at 25°C with different molar ratio of Affitins (Figure 5B). A ratio-dependent inhibition of activity was observed for the three binders. The K_i_ for the anti-CelD binders was determined as 111 nM for H3 and 95 nM for E12, while for the anti-HEWL binder a K_i_ value of 45 nM was obtained (Table 1). This latter value is similar to that observed for the HEWL-specific camel V_H_H antibody (50 nM) [11], [15]. The differences between the K_D_ and K_i_ values for Affitin H4 could result from experimental errors associated with the measurement of cell lysis for K_i_ determination.

CelD is a thermophilic glycosidase from *Clostridium thermocellum* and its optimal temperature for catalysis is 60°C [38]. As H3 and E12 Affitins are thermostable, it was possible to show that their inhibition properties at 60°C (Figure 5B) were similar to those determined at 25°C.

### Crystal Structures of Affitin-enzyme Complexes

**Figure 8:**
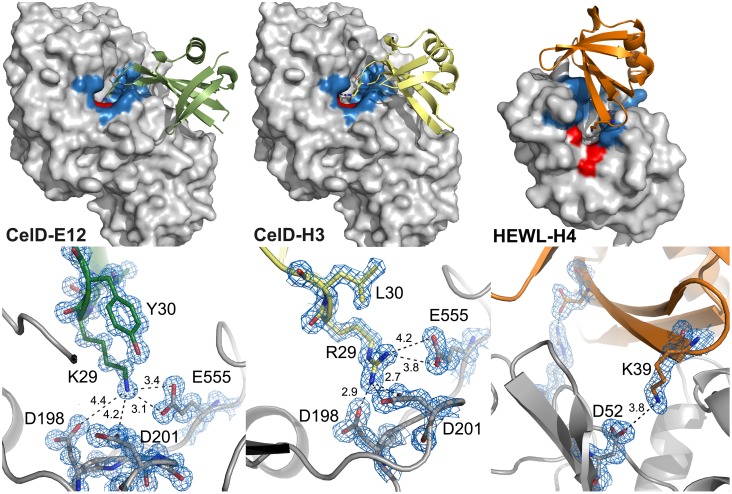
Crystal structures of anti-glycosidase Affitins in complex with their targets. Glycosidases are represented as gray surfaces with catalytic clefts colored in blue and catalytic residues in red. Affitins are represented in cartoons. The bottom panel shows a close-up view of the contacts and distances (Å) of the catalytic residues involved in salt-bridges and H-bonds (discontinuous lines). In blue, σA-weighted 2*mFobs* - *DFcalc* electron-density map contoured at 1.2 sigma for the HEWL-H4 complex, and at 2.0 sigma for CelD-H3 and CelD-E12 complexes. Residues and bond distances are indicated.

To analyze interactions at the atomic level, the crystal structures of the CelD-E12, CelD-H3 and HEWL-H4 complexes were determined at 1.1, 1.6 and 1.5 Å resolution, respectively (Figure 8). All complexes were crystallized in different crystal forms and the structures were solved by molecular replacement techniques using available enzyme structures as search models. Data collection and refinement statistics are presented in Table 2. Neither CelD nor HEWL structures underwent significant conformational changes upon Affitin binding, with RMSDs between Affitin-bound and ligand-free enzyme structures are 0.292 and 0.171 Å, respectively. Despite the large number of mutations and insertions in Sac7d, the overall fold was preserved in the three Affitins, *i.e.* an SH3-like five-stranded incomplete β-barrel capped by a C-terminal α-helix. As previously noticed with an anti-human IgG Affitin [23], the conserved β-barrel core did not show significant deviations when compared with the X-ray structure of wild-type Sac7d PDB code: 1AZP (RMSD <0.45 Å). Interestingly, we expected from our library designs a loop of 6 residues in length for H3 and E12 Affitins; however, it was partly structured in both cases by the extension of β3- and β4-strands (Figure 4). Calculated shape complementarity (Sc) values for each complex were 0.75, 0.72 and 0.76 for HEWL-H4, CelD-E12 and CelD-H3, respectively. These values are in agreement with those obtained by Lawrence and Colman [36] for protein/protein inhibitor interfaces (0.70–0.76), whereas for antibody/antigen interfaces Sc values are usually between 0.64–0.68.

**Table 2:** Data collection and refinement statistics.

	HEWL-H4	CelD-E12	CelD-H3
**Data collection:**			
Resolution range (Å)	43.04–1.5 (1.53–1.5)	48.71–1.1 (1.12–1.1)	46.79–1.63 (1.66–1.63)
Space group	P2_1_	P4_3_	P2_1_2_1_2_1_
Unit cell			
a, b, c (Å)	37.89, 62.82, 87.11	87.63, 87.63, 97.42	74.42, 97.73, 106.58
α, β, γ (°)	90, 98.7, 90	90, 90, 90	90, 90, 90
R-sym	0.036 (0.347)	0.109 (0.751)	0.053 (0.359)
R-meas	0.051 (0.491)	0.134 (0.916)	0.069 (0.473)
No. of unique reflections	62318 (3035)	292047 (14154)	96777 (4638)
I/σ(I)	16.9 (2.8)	10.5 (2.9)	15.4 (3.2)
Completeness (%)	96.3 (94.3)	98.4 (96.3)	99.7 (96.9)
Multiplicity	3.5 (3.4)	5.8 (5.7)	4.0 (3.9)
CC ½	0.998 (0.853)	0.997 (0.759)	0.998 (0.857)
**Refinement:**			
Resolution (Å)	1.50	1.10	1.63
No. of reflections	62318	277269	96678
R-factor/R-free	0.13/0.17	0.10/0.12	0.11/0.14
No. of atoms			
Macromolecules	3001	4711	4838
Ligands/ions	12	40	28
Water	358	866	781
RMS bonds (Å)	0.020	0.026	0.020
RMS angles (°)	1.884	2.030	1.814
**B-factor (Å^2^):**			
Macromolecules	27.0	12.0	18.2
Ligands/ions	34.0	13.6	24.5
Water	36.3	27.8	32.8

Statistics for the highest-resolution shell are shown in parentheses.

#### Structural analysis of the interaction of the CelD-anti-CelD Affitins

E12 and H3 Affitins in complex with CelD displayed a similar interaction with an average buried surface area of 1317 Å^2^ and an Affitin contribution of 707 and 717 Å^2^, respectively (Figure 8, Table 3). In both cases, the Affitins bound the enzyme by inserting the protruding extended loop into the active site while the β-sheet 2 rested on the CelD surface. Additionally, the loop presented a charged residue that interacted *via* salt-bridges with those involved in the catalytic reaction.

**Table 3:** Interaction analysis of anti-glycosidase Affitins.

Affitin	Randomized region	H-bonds^a^^,^ b	Salt-bridges^a^	Hydrophobic contacts^b^	Affitin BSA^a^	Complex BSA^a^
H4	Surface	11	2	10	838.7	1749.7
H3	Surface + loop	6	6	11	717.5	1317.3
E12	Surface + loop	5	2	7	707.1	1316.7

a Interaction contacts analyzed with the PISA server.

b Data obtained with Protein Interactor Calculator at 5 Å cutoff.

BSA: Buried surface area (Å^2^), calculated with a water probe of 1.4 Å diameter.

The structure of the CelD-E12 complex suggested that Lys29 was the main residue responsible for binding and activity inhibition. It formed a salt-bridge through its nitrogen NZ with Glu555 of CelD, the residue that acts as a proton donor in catalysis [39]. Other key interactions involved E12 residues Gln35, Arg50 and Arg46 (non-randomized position), which were hydrogen-bonded to residues Tyr455, Tyr551 and Pro539 of CelD. These latter interactions stabilized the complex by positioning the β-sheet 2 over the CelD surface. In addition, due to Tyr551, which is positioned at the external part of the active site, the interaction with Arg50 limited access to the substrate even more. Finally, Lys29 also formed H-bonds *via* the main chain with Glu353 and Tyr354, which fix the artificial loop into the enzyme cavity.

For the CelD-H3 complex (Figure 8), the overall positioning of the Affitin onto CelD was similar. However, some contacts were different from those observed for the CelD-E12 complex. For example, Arg29 formed salt-bridges with other catalytically important residues (Asp198 and Asp201) [39], [40].

#### Structural analysis of the interaction of the HEWL-anti-HEWL Affitins

The structure of Affitin H4 in complex with HEWL showed an unusual mechanism of inhibition (Figure 8). Unlike V_H_H domains or anti-CelD Affitins, the randomized flat surface interacted with the enzyme by covering the catalytic site. A total buried surface area of 1750 Å^2^ resulted from this interaction, to which Affitin H4 contributed 839 Å^2^. This binding interface is larger than Affitins with the extended loop. There were seven residues from Affitin H4 involved in H-bonds, including the non-randomized Val23 and Gly38. Hydrophobic residues were found which spatially complement the interaction interface, especially residues Trp8, Trp23, and Tyr43 which are located inside the catalytic site, filling the cleft. These aromatic residues have a dual role forming intra- (Tyr43) and intermolecular (Trp8 and Trp23) H-bonds. Residues Lys39 and Asp44 formed two salt-bridges, which sealed the catalytic site by anchoring at the β5-strand ends of Affitin H4. The non-randomized Lys39 formed a salt-bridge with residue Asp52, which acts as a nucleophile to generate a glycosyl enzyme intermediate that is critical for HEWL activity. The interactions observed confirmed our previous results obtained by mutagenesis scanning of Affitin H4 [24].

## Discussion

In this study, we demonstrate that specific and potent inhibitors of two glycosidases with at least two modes of binding can be derived from a unique scaffold protein. Catalytic residues are usually positioned inside a substrate-binding cleft or pocket on the enzyme’s surface, and therefore molecules capable of binding deep inside these cavities or covering them can represent invaluable tools for glycosidase inhibition.

Small-molecule inhibitors are usually the preferred choice when targeting glycosidases, due to their pharmacological properties and because they can fit inside catalytic sites. About 1% of the human genome encodes for glycosyl processing enzymes [41], and among these 300 enzymes, 90 are glycosidases according to the CAZy database [42]. It is not ideal that small inhibitors mainly interact with catalytic residues often conserved among different glycosidases. Combining a high specificity and potency in one small molecule is thus difficult to achieve [6], [8].

Proteinaceous inhibitors can bind to enzymes *via* a large surface area and are not limited to cavities. This enables them to interact with residues from non-conserved regions on the target, making this class of inhibitors potentially more specific. Artificially-generated inhibitors based on protein scaffolds are attractive since their properties, such as molecular weight, stability, lack of disulfide bridges or ease of production, can be chosen. In order for this approach to be generalized with minimal development effort, it is crucial that the same scaffold can bind to the different cavity shapes found in enzymes. With the design of several libraries and exploiting the high plasticity of the Sac7d scaffold, we were able to program different modes of binding in Affitins [23]. Here, we randomized a surface on Sac7d and extended the loop 2 with the aim of gaining loop flexibility and a potential to bind clefts. Using these different libraries, we obtained thermally stable binders with high affinity in the nanomolar range and specificity for thermostable CelD and for HEWL.

All three Affitins were shown to be inhibitors of two evolutionary distant endo-glycosidases, which both hydrolyze the O-glycosyl bond and have cleft-shaped catalytic sites but use two different enzymatic mechanisms. These anti-glycosidase Affitins have a K_i_ in the nanomolar range, which makes them comparable to the few best glycosidase inhibitors available that have a K_i_ ∼10^−9^ to 10^−8^ M [25]. Thus, we have obtained potent inhibitors with no cross-recognitions as shown by ELISA and ITC analysis. These Affitins are efficient inhibitors even at high temperatures (at least 60°C) although selected at 4°C. These could be useful as basic research tools to study *in vivo* biological events in thermophilic micro-organisms.

We have solved the crystal structure of the different complexes at high resolution, which shows that the recognized epitope is located in the catalytic cleft for both targets. The enzymatic inhibition properties are thus explained by hindrance of substrate access. Furthermore, the structures reveal that there is a direct interaction by H-bonds and salt-bridges with catalytically important residues in both enzymes, thereby locking the catalytic activity. The buried surfaces of the complexes (from 1317 Å^2^ to 1749 Å^2^) are comparable to natural protein-protein interactions [10]. Studies with other scaffolds have reported a modulation of the recognition by mutagenesis on their surface, on loop(s) or both [12], [13], [43]. Here, we present library designs providing Affitins using two modes of binding in an independent way, as shown by the structures of the complexes: by β-sheet 2 surface (Affitin H4), and a combination of β-sheet 2 surface and loop 2 (Affitins H3 and E12). E12 and H3 Affitins, which are derived from libraries with a longer and randomized loop 2, present a protruding convex region that penetrates the catalytic cleft of CelD, thereby validating our strategy to use an extended randomized loop. These structural data expand the possibilities of designing binding surfaces on Sac7d capable of recognizing different topographies in protein targets. They also provide useful hints for further inhibitor improvements, for example by randomizing residues that were kept constant in our library designs while they were identified in this work as interacting with targets. Importantly, no screen for enzymatic inhibition was performed to isolate the three Affitins that bind in two different catalytic sites. It remains to be seen if this is general but we believe this is not a fortuitous result, and suggests that Affitins have a propensity to bind where the curvature of the protein surface changes. In addition, the structures of Affitin-glycosidase complexes highlight that Affitins bind not only to catalytic-site residues but also to surrounding residues, contributing to their specificity. Variable domains of heavy-chain shark and camel anti-HEWL antibodies have been selected and structurally characterized [11], [15], [16], [17]. Some of these were found to inhibit lysozyme activity by a mechanism similar to that reported here for anti-CelD Affitins. For instance, the CDR3 from a shark V-NAR was shown to be inserted into the HEWL active site and to engage in a salt-bridge interaction with the HEWL catalytic residue Asp52 [17].

For research or clinical purposes, it is important that the inhibitor does not interact with other glycosidases from the same organism of interest. We thus analyzed the alignment of sequences of all seventeen *C. thermocellum* (ATCC 27405) glycosidases from the GH9 CAZy family (including EC 3.2.1.4, EC 3.2.1.151, EC 3.2.1.91 enzymes) to which CelD belongs. We observed that among the residues of CelD interacting with Affitin E12 (Glu353, Tyr354, Val357, Tyr455, Trp538, Pro539, Tyr551, Glu555), three residues were not found in other glycosidases (Glu353, Val357, Trp538), while Pro539 was found in only three other glycosidases, suggesting a high specificity of E12. Furthermore, all these residues are outside or at the edge of the CelD catalytic site, confirming that E12 can recognize residues surrounding a catalytic site. We believe that such inhibitors might be a good starting point for the design of a new generation of low molecular weight drugs to modulate the activity of the most challenging targets in pharmaceutical research.

Protein-based therapeutics have been shown to be successful in clinical use and while monoclonal antibodies represent ∼48% of these commercial recombinant proteins [44], [45], there are also examples of non-human proteins, such as hirudin, which are used as therapeutics [46]. Given the difficulties related to antibody production, alternative scaffolds have recently been developed as binding molecules. Furthermore, several artificial affinity proteins with inhibition properties (for a review, see ref. [13]) derived from alternative scaffolds are undergoing clinical trials in the phases II/I [47] with the aim of using them as therapeutics. These include the Kunitz domain [48], [49], Adnectin [50], and DARPins [51]. Monobodies are another source of binders that have been engineered to generate inhibitor molecules [52], [53]. These alternative proteins present one or several attractive features, such as high-level expression in bacteria in soluble form, a simple monomeric structure, and stability toward denaturing agents and temperature. Although the performance of our Affitin-based class of inhibitors is yet to be evaluated *in vivo*, as demonstrated in the present and previous works, Affitins can be used as artificial binders and contain all these features with the additional property of resisting a wide pH range (usually from 0 to at least 10 and up to pH = 13). This combination of favorable properties and the resistance of Sac7d to harsh acidic conditions [23] may be interesting to inhibit targets within the digestive tract which are associated with pathologies such as α-glucosidase and diabetes type II [7].

We have previously described Affitins capable of inhibiting the type II secretion system (T2SS) in bacteria [19]. Here, we propose a strategy for generating potent glycosidase inhibitors with different modes of binding. We anticipate that Affitin-based inhibitors are not limited to glycosidases and may represent a generic method to obtain specific enzyme inhibitors with favorable properties interesting for research and clinical applications, and may provide an innovative approach for drug discovery.
